# Effect of noninvasive ventilation on mortality and clinical outcomes among patients with severe hypoxemic COVID-19 pneumonia after high-flow nasal oxygen failure: a multicenter retrospective French cohort with propensity score analysis

**DOI:** 10.1186/s12931-024-02873-4

**Published:** 2024-07-15

**Authors:** Antoine Goury, Zeyneb Houlla, Mathieu Jozwiak, Tomas Urbina, Matthieu Turpin, Alexandra Lavalard, Driss Laghlam, Sebastian Voicu, Jeremy Rosman, Claire Coutureau, Bruno Mourvillier

**Affiliations:** 1 Unité de Médecine Intensive et Réanimation Polyvalente, CHU Reims, Reims, F-51100 France; 2 Service de Médecine Intensive Réanimation, Centre Hospitalier Universitaire de Nice, Hôpital l’Archet 1, Nice, France; 3 UR2CA - Unité de Recherche Clinique Côte d’Azur, Université Côte d’Azur, Nice, France; 4 Service de Médecine Intensive Réanimation, Hôpital Saint-Antoine, Assistance Publique Hôpitaux de Paris, Paris, France; 5 Service de Médecine Intensive Réanimation, Hôpital Tenon, Assistance Publique Hôpitaux de Paris, Paris, France; 6 Unité de Réanimation Polyvalente, Centre Hospitalier de Troyes, Troyes, France; 7 Service de Médecine Intensive et Réanimation, Hôpital Cochin, Assistance Publique Hôpitaux de Paris, Paris, France; 8 Réanimation Médicale et Toxicologique, Hôpital Lariboisière, Assistance Publique Hôpitaux de Paris, Paris, France; 9 Unité de Soins Intensifs et Réanimation, Groupe Hospitalier de Territoire Nord-Ardennes, Charleville-Mézières, France; 10 Université de Reims Champagne-Ardenne, VieFra, Reims, F-51100 France; 11 Unité d’Aide Méthodologique, CHU Reims, Reims, F-51100 France; 12 Université de Reims Champagne-Ardenne, EA-4684 CardioVir, Reims, F-51100 France

**Keywords:** COVID-19, Acute respiratory failure, Noninvasive ventilation, High flow oxygen therapy, Outcomes, Mortality, Intubation, Acute respiratory distress syndrome

## Abstract

**Background:**

We assessed the effect of noninvasive ventilation (NIV) on mortality and length of stay after high flow nasal oxygenation (HFNO) failure among patients with severe hypoxemic COVID-19 pneumonia.

**Methods:**

In this multicenter, retrospective study, we enrolled COVID-19 patients admitted in intensive care unit (ICU) for severe COVID-19 pneumonia with a HFNO failure from December 2020 to January 2022. The primary outcome was to compare the 90-day mortality between patients who required a straight intubation after HFNO failure and patients who received NIV after HFNO failure. Secondary outcomes included ICU and hospital length of stay. A propensity score analysis was performed to control for confounding factors between groups. Exploratory outcomes included a subgroup analysis for 90-day mortality.

**Results:**

We included 461 patients with HFNO failure in the analysis, 233 patients in the straight intubation group and 228 in the NIV group. The 90-day mortality did not significantly differ between groups, 58/228 (25.4%) int the NIV group compared with 59/233 (25.3%) in the straight intubation group, with an adjusted hazard ratio (HR) after propensity score weighting of 0.82 [95%CI, 0.50–1.35] (*p* = 0.434). ICU length of stay was significantly shorter in the NIV group compared to the straight intubation group, 10.0 days [IQR, 7.0-19.8] versus 18.0 days [IQR,11.0–31.0] with a propensity score weighted HR of 1.77 [95%CI, 1.29–2.43] (*p* < 0.001). A subgroup analysis showed a significant increase in mortality rate for intubated patients in the NIV group with 56/122 (45.9%), compared to 59/233 (25.3%) for patients in the straight intubation group (*p* < 0.001).

**Conclusions:**

In severely hypoxemic COVID-19 patients, no significant differences were observed on 90-day mortality between patients receiving straight intubation and those receiving NIV after HFNO failure. NIV strategy was associated with a significant reduction in ICU length of stay, despite an increase in mortality in the subgroup of patients finally intubated.

**Supplementary Information:**

The online version contains supplementary material available at 10.1186/s12931-024-02873-4.

## Background

The coronavirus disease 2019 (COVID-19) pandemic led to a significant increase in patients admitted in intensive care units (ICUs) for acute respiratory failure [1]. This outbreak highlighted the limitations of healthcare systems, particularly in ventilators and ICU beds availability. It was therefore urgent to identify the best oxygenation strategies to avoid invasive mechanical ventilation (IMV) and its associated complications [2]. High-flow nasal oxygen (HFNO) has been recommended as the first-line treatment for acute hypoxemic respiratory failure and has been widely applied in patients with COVID-19 [3, 4]. Consequently, numerous trials have investigated the benefits of HFNO and alternative oxygenation supports including standard oxygen therapy, noninvasive ventilation (NIV) and continuous positive airway pressure (CPAP) with conflicting results on intubation and mortality [5–11]. Among these studies, noninvasive ventilation strategies, notably NIV with helmet and CPAP, could reduce the need for intubation and mortality among patients with moderate to severe COVID-19 hypoxemic respiratory failure [5, 10]. However, noninvasive respiratory strategies, by delaying the time of intubation, could expose patients to more adverse events [6, 10].

Thus, the timing of intubation remains debated and final decision on intubation is always left to the physician in charge. Some authors argue for an “early intubation” strategy to limit self-inflicted lung injury (P-SILI) and pulmonary sequelae [12, 13], with the risk of overflowing ICU beds. Others support a “wait and see” approach with noninvasive respiratory strategies [14–16], with the risk of late failure and increased mortality.

Given the lack of consensus, we believe that further research is important to determine for which patients, noninvasive strategies have a positive effect on clinical outcomes. On this basis, this retrospective multicenter observational study had the following objectives: (1) to assess the effect of additional NIV on 90-days mortality in critically ill COVID-19 patients after HFNO failure; (2) to determine the effect of this strategy on ICU and hospital length of stay; (3) a subgroup analysis of clinical outcomes and adverse events among intubated patients and risk factors associated with NIV failure.

## Methods and patients

### Study design and settings

This retrospective observational multicenter cohort study was conducted in 5 university-affiliated hospital ICUs and in 2 non-affiliated hospital ICUs in France **(Additional file 1: Table ****S1****)**. This study was performed in compliance with the national legislation regarding observational retrospective studies and declared at European General Data Protection Regulation (declaration N°:MR00408112021). In accordance with national ethical directives, the requirement for written informed consent was waived. According to the French Public Health Code, an Institutional Review Board was not necessary for this research. This retrospective study report complies with the Strengthening the Reporting of Observational Studies in Epidemiology (STROBE) Statement guidelines **(Additional file 2; Table S2**).

### Patient selection

Consecutive patients with acute hypoxemic respiratory failure and a positive COVID-19 PCR-test admitted in participating ICUs were screened for inclusion. Patients were eligible if the following inclusion criteria were met: aged ≥ 18 years, treated with dexamethasone according to guidelines and received HFNO as the first-line treatment [4]. Only patients with a HFNO failure were included in the final analysis. Exclusion criteria were as follows: patients who received NIV as the first-line oxygenation support, patients who received HFNO or NIV for less than one hour, patients previously included in a trial on oxygen support strategies, patients with a decision of withdrawal of life-sustaining therapy, patients who died within the 48 h after ICU admission, medical records with missing data concerning HFNO, NIV or intubation management, lost to follow up or transfer in a non-participating center.

### Oxygenation strategy and treatment failure

In all participating hospitals, HFNO was administered all day long until recovery or initiation of IMV or NIV. NIV was applied for at least 1 h or continuously according to patient’s tolerance. HFNO was applied between NIV sessions. Any modification in ventilator settings and interface setup to optimize patient-ventilator interaction was left to the discretion of the attending physicians.

HFNO failure was defined by the physician’s decision to intubate or to switch from HFNO to NIV. This decision was based on the usual standard of care for endotracheal intubation [9, 17]. Switching from HFNO to NIV was based on signs of persistent or worsening respiratory failure with desaturation despite 100% FIO2 HFNO or intolerance/agitation. Throughout the study, intubation or switch to NIV was left to the physician’s discretion and was not standardized for all centers. To minimize this bias, a dedicated study board (AG and ZH) reviewed a posteriori the medical records and verified whether that decision met the required criteria.

### Measurement

Collected data included SAPSII, SOFA, time from onset of symptoms to hospital and ICU admission, duration of dexamethasone treatment, the percentage of pulmonary infiltrate from the first CT-scan performed after ICU admission, respiratory parameters including the ROX (respiratory rate oxygenation) index at H2 under HFNO. The first blood gas under HFNO was recorded as soon as possible within 12 h of the introduction of HFNO. The last blood gas under HFNO was recorded just before intubation or switch to NIV or within the last 12 h under HFNO. Duration of HFNO, NIV and IMV were also reported. In the NIV group, the duration of HFNO also included intermittent HFNO between NIV sessions. For intubated patients in each group, following clinical outcomes or adverse events were collected; cardiac arrest, renal replacement therapy, pulmonary embolism, ventilator-associated pneumonia (VAP), barotrauma, prone positioning, nitric oxide use, neuromuscular blockade, extracorporeal membrane oxygenation.

### Outcomes

The primary outcome of the study was the 90-day mortality in patients who received a straight intubation after HFNO failure (straight intubation group) and in patients who received NIV after failed HFNO (NIV group). Secondary outcomes were the ICU and hospital length of stay. Exploratory outcomes included; a subgroups analysis (straight intubation, non-intubated NIV and intubated NIV groups) for the 90-day mortality and length of stay; to identify risk factors for NIV failure; to report clinical outcomes and adverse events for intubated patients in each subgroups.

### Statistical analysis

Categorical variables were expressed as number with percentage (%) and continuous variables as median with interquartile range (IQR). Initial characteristics of the “straight intubation group” and the “NIV group” were compared using a Chi-square test or Fisher’s exact test for the categorical data and a Mann-Whitney U for continuous data.

The effect of NIV on 90 days mortality and the hospital and ICU length of stay was assessed using a propensity score analysis to control confounding factors between groups. Covariates included in the propensity score model were confounders and variables related to the outcome (i.e. 90 days mortality) [18]. Thus, the model included the following comorbidities; sex, age, body mass index (BMI), hypertension, diabetes, chronic heart failure, chronic respiratory diseases, chronic renal failure, immunocompromised state and solid cancers. Moreover, severity markers such as the SAPS II score at admission, the percentage of pulmonary infiltrate on the first chest CT-scan, the ROX index at H2 with HFNO and the last PaO2/FiO2 ratio before intubation or switch to NIV were also included. Then, a weighted logistic regression with stabilized inverse probability of treatment weighting (IPTW) was performed, adjusted for center. A sensitivity analysis was performed with propensity score matching on a 1:1 ratio using a caliper of 0.1. To deal with missing data, multiple imputations by chained equations were performed [19]. Standardized mean difference method was used to examine the balance of covariate distribution between treatment groups before and after weighting **(Additional file 3: Fig ****S1****).**

In secondary endpoints, we investigated the length of stay in ICU and hospital between groups using a weighted cause-specific proportional hazards model performed on the imputed dataset. Patients who died before ICU or hospital discharge were censored at the time of death.

We also explored risk factors associated with NIV failure in bivariate analyses. Quantitative variables were then dichotomized using a threshold corresponding to a sensitivity of at least 80% to predict NIV failure in order to better stratify patients at risk of intubation. A p-value < 0.05 was considered statistically significant. Additional exploratory analyses were performed on the clinical outcomes in subgroups. All analyses were performed using R software, version 4.1.2 (R Core Team (2023) R Foundation for Statistical Computing, Vienna, Austria).

## Results

**Fig. 1:**
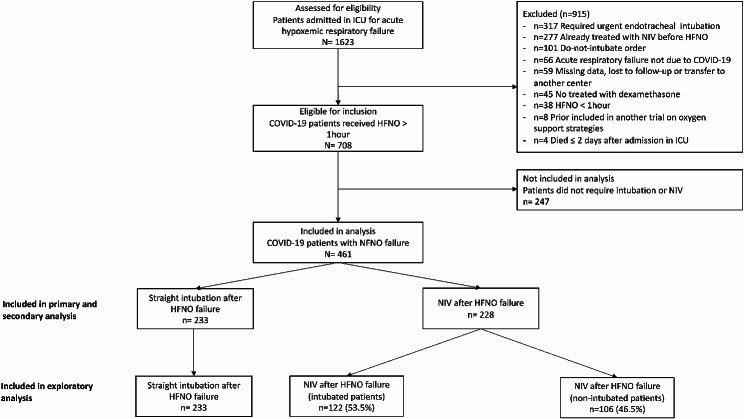
Flow chart ICU: intensive care unit, HFNO: high-flow nasal oxygen, NIV: noninvasive ventilation

Between December 2020 to January 2022, 461 patients with a severe COVID-19 pneumonia and HFNO failure were included in the analysis. Among them, 228 patients received NIV and 233 patients received straight intubation after HFNO failure **(**Fig. 1**)**. NIV was performed alternately with HFNO for *n* = 178/228 (78%) of the patients included.

Patients characteristics at baseline.

Participants experienced a similar severe hypoxemic respiratory failure at initiation of HFNO with a median PaO2/FiO2 ratio of 86 [67–110] and 88 [70–119] for straight intubation and NIV group respectively (*p* = 0.288).

Patients in the NIV group had less solid cancers 14/228 (6%) than patients in the straight intubation group 32/233 (14%) (*p* = 0.007), had a significantly higher median BMI, 31 kg.m^−^² [IQR,27–35] vs. 28 kg.m^−^² [IQR,26–32] (*p* < 0.001) and were more hypercapnic at baseline with median PaCO2 of 35 mmHg [IQR,32–38] vs. 33 mmHg [IQR,29–37] (*p* = 0.002).

Regarding ICU severity scores, patients in the straight intubation group were more severely ill than patients who received NIV with a median SOFA and SAPSII score of 4 [IQR,3–6] vs. 4 [IQR,3–5] (*p* < 0.001) and 38 [IQR,31–49] vs. 31 [IQR,25–37], respectively (*p* < 0.001). They also had more signs of worsening respiratory failure at H2 under HFNO compared to the NIV group with a median respiratory rate of 29/min [IQR,25–34] vs. 26/min [IQR,22–31] (*p* < 0.001) and a lower ROX index of 3.8 [IQR,3.2–5.2] vs. 4.5 [IQR,3.5–5.6] (*p* = 0.002). Finally, patients who received straight intubation had the worst PaO2/FiO2 ratio on the last arterial blood gas before HFNO failure compared to the NIV group 71 [IQR,60–87] vs. 88 [IQR,71–112] (*p* < 0.001). The other baseline patient’s characteristics are summarized in Table 1. The reasons why practitioners intubated patients or switched from HFNO to NIV are shown in **Additional file 4: Table S3.**

**Table 1:** Baseline patients characteristics

	All patients (*N* = 461)	Straight intubation after HFNO failure (*n* = 233)	NIV after HFNO failure (*n* = 228)	*p*-value	Missing data (*n*)
Age. median [IQR], y	64 [55;72]	66 [55;72]	63 [55;72]	0.496	-
Males, n (%)	317 (69)	161 (69)	156 (68)	0.875	-
BMI. median [IQR], kg.m^−^²	29 [26;33]	28 [26;32]	31 [27;35]	< 0.001	-
SOFA score at admission, median [IQR]	4 [3;5]	4 [3–6]	4 [3–4]	< 0.001	-
SAPSII, median [IQR]	35 [27–44]	38 [31–49]	31 [25–37]	< 0.001	-
Time from, median [IQR], d					
Symptoms onset to Hospital admission	7 [5–9]	7 [5–9]	7 [5–9]	0.404	-
Time from ICU to treatment (straight intubation or NIV)	1 [0–2]	1 [0–2]	1 [0–2]	0.451	2
HFNO duration^a^, d	2 [1–6]	1 [0–2]	5 [2–8]	< 0.001	3
Comorbidities, n (%)					
Hypertension	248 (54)	120 (52)	128 (56)	0.318	-
Diabetes mellitus	164 [36]	80 [34]	84 [37]	0.574	-
Chronic heart failure^b^	77 [17]	36 [16]	41 [18]	0.466	-
Chronic lung disease^c^	61 [13]	33 [14]	28 [12]	0.551	-
Chronic kidney disease^d^	34 [7]	21 [9]	13 [6]	0.174	
Immunocompromised state^e^	54 [12]	32 [14]	22 [10]	0.173	-
Solid cancer	46 [10]	32 [14]	14 [6]	0.007	-
First chest CT scan findings					
Percentage of pulmonary infiltrates, median [IQR]	60 [37–66]	50 [37–62]	60 [37–70]	0.540	29
Pulmonary embolism, n (%)	13 [3]	4 [2]	9 [4]	0.129	28
Respiratory findings at H2 of HFNO, median [IQR]					
Oxygen saturation, %	94.0 [92.0-96.1]	94.0 [92.0-96.6]	94.0 [92.0–96.0]	0.959	15
FiO2, %	87.5 [70.0-100.0]	90 [70–100]	80 [70–100]	0.148	17
Respiratory rate, breaths/min	28 [23–33]	29 [25–34]	26 [22–31]	< 0.001	46
ROX index	4.1 [3.3–5.3]	3.8 [3.2–5.2]	4.5 [3.5–5.6]	0.002	49
Positive end-expiratory pressure at initiation (cmH2O) First arterial blood gas under HFNO^f^, median [IQR]	-	-	6 [5–7]	-	-
FiO2, %	80 [60–100]	80 [60–100]	80 [60–100]	0.875	12
PaO2, mmHg	71 [61–86]	71 [61–85]	72 [61–89]	0.757	28
PaCO2, mmHg	34 [31–38]	33 [29–37]	35 [32–38]	0.002	28
PaO2/FiO2 ratio	87 [69–113]	86 [67–110]	88 [70–119]	0.288	30
Last arterial blood gas under HFNO^g^, median [IQR]					
FiO2, %	95 [80–100]	100 [80–100]	90 [75–100]	< 0.001	53
PaO2, mmHg	66.8 [57.0-82.2]	65 [55–76]	71 [60–89]	< 0.001	46
PaCO2, mmHg	33.6 [30.0-37.2]	33 [29–37]	35 [32–38]	< 0.001	36
PaO2/FiO2 ratio	78.5 [63.0-100.0]	71 [60–87]	88 [71–112]	< 0.001	54

Abbreviations: ICU: intensive care unit. HFNO: high flow nasal oxygenation. NIV: noninvasive ventilation. BMI: body mass index. SOFA: sequential organ failure assessment. SAPSII: simplified acute physiology score II. ROX index defined as (SpO2/FiO2)/respiratory rate

^a^ HFNO before intubation or NIV was administrated continuously. In the NIV group, HFNO was administrated between NIV sessions for patients receiving intermittent NIV (*n* = 187)

^b^ Chronic heart failure corresponds to history of coronary artery disease. or documented heart failure in the medical records

^c^ Chronic obstructive pulmonary disease or obstructive sleep apnea

^d^ Chronic kidney disease corresponds to a KDIGO stage 3 and more

^e^ Immunocompromised state corresponds to patients with hematologic malignancies, treatment based on corticosteroids or immunosuppressive therapies

^f^ the closest to the introduction of the HFNO and within 12 h maximum

^g^ the closest to the intubation or switch to NIV and within the 12 last hours with HFNO.

Primary outcome.

**Fig. 2:**
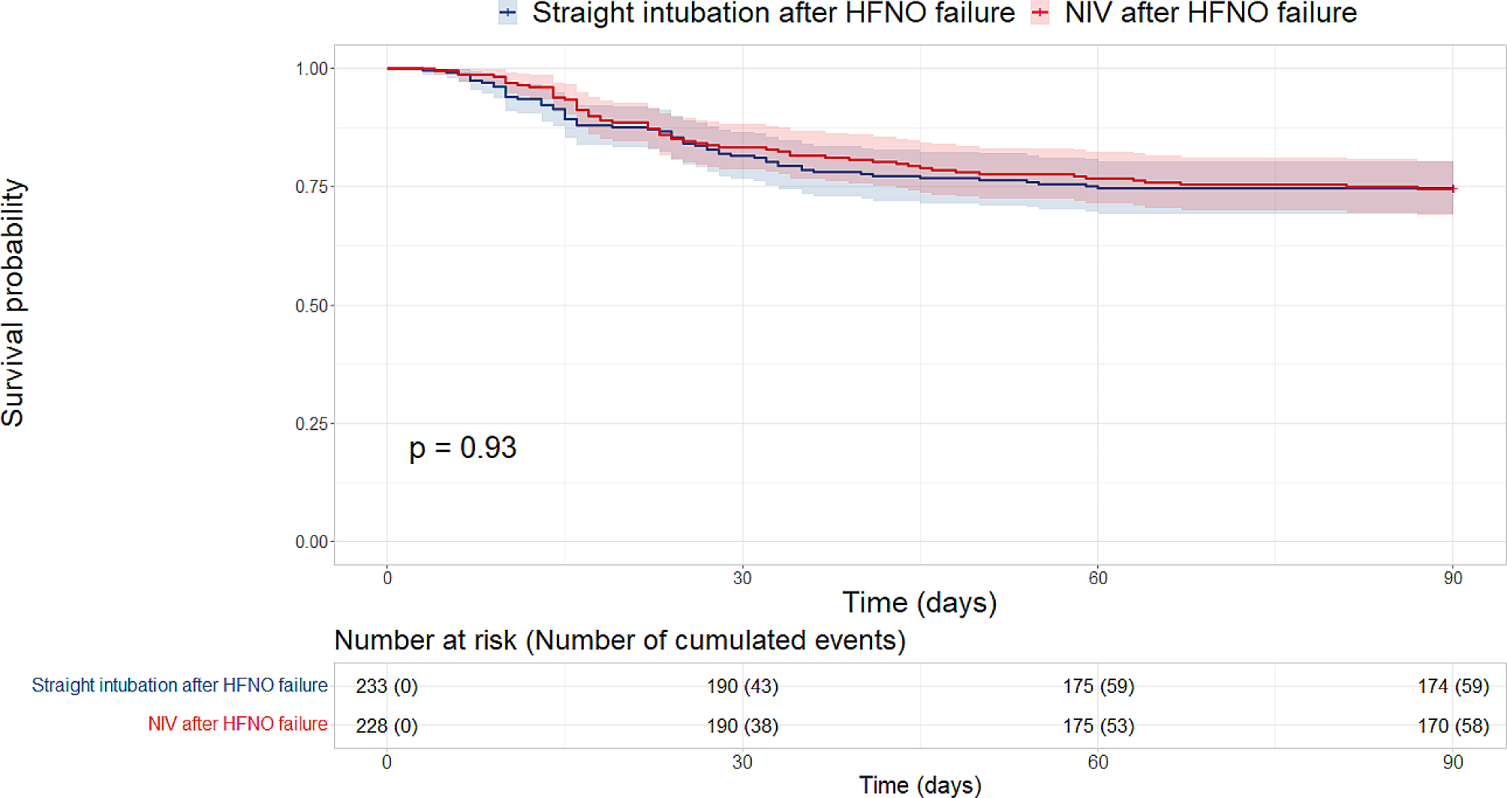
Comparison of Kaplan-Meier survival curves between straight intubation after HFNO failure group and NIV after HFNO failure group. Abbreviations: HFNO: high flow nasal oxygenation. NIV: noninvasive ventilation

The 90-day mortality did not significantly differ between groups, 58/228 (25.4%) int the NIV group compared with 59/233 (25.3%) in the straight intubation group, with an adjusted hazard ratio (HR) after propensity score weighting of 0.82 [95%CI, 0.50–1.35] (*p* = 0.434). A sensitivity analysis with propensity score matching found similar results with a HR of 0.75 [95%CI, 0.41–1.39] (*p* = 0.358) **(**Tables 2**and** Fig. 2).

**Table 2:** Primary and secondary outcomes according to study group before and after propensity score weighting

Outcomes	Straight intubation after HFNO failure	NIV after HFNO failure	Unadjusted		Propensity score weighting	
			HR [IC95%]	*p*-value	HR [IC95%]	*p*-value
**Primary outcome**						
Mortality at Day-90, No./total (%)	59/233 (25.3)	58/228 (25.4)	0.98 [0.68–1.41]	0.928	0.82 [0.50–1.35]	0.434
**Secondary outcomes** ^**a**^						
Length of stay, median [IQR], d						
In ICU	18.0 [11.0–31.0]	10.0 [7.0-19.8]	1.26 [1.02–1.55]	0.03	1.77 [1.29–2.43]	< 0.001
In Hospital^b^	28.0 [17.0–44.0]	19.0 [14.0–33.0]	1.22 [0.98–1.50]	0.07	1.96 [1.41–2.71]	< 0.001

Abbreviations: HFNO: high flow nasal oxygenation. NIV: noninvasive ventilation. ICU: intensive care unit

^a^ Secondary outcomes were assessed only in patients alive at Day-90 (*n* = 344)

^b^ One missing data (*n* = 343)

Secondary outcomes.

Among patients alive at hospital discharge (*n* = 343) length of stay in ICU was significantly shorter in the NIV group compared to the straight intubation group, 10.0 days [IQR, 7.0-19.8] versus 18.0 days [IQR,11.0–31.0] respectively. The weighted Cox proportional hazards models showed a HR of 1.77 [95%CI, 1.29–2.43] (*p* < 0.001). Length of stay in hospital was also significantly shorter in the NIV group with 19 days [14–33] vs. 28 days [17–44] in the straight intubation group, with a HR of 1.96 [95%CI, 1.41–2.71] (*p* < 0.001) **(**Table 2**)**.

Exploratory outcomes.

Exploratory analyses showed a significant difference in 90-day mortality between intubated patients in the NIV group with 56/122 (45.9%), compared to 2/106 (1.9%) for non-intubated patients in the NIV group and 59/233 (25.3%) for patients in the straight intubation group (*p* < 0.001) (Table 3, **Additional File 5: Fig S2**). Among patients alive at hospital discharge, the length of stay in ICU was 18 days [IQR, 11–31] in the straight intubation group, 21.5 days [IQR, 13.0–32.0] for intubated patients in the NIV group and 8.0 days [5.0-10.2] for non-intubated patients in the NIV group (*p* < 0.001).

**Table 3:** Exploratory outcomes: unadjusted subgroup analysis for mortality and length of stay

	Straight intubation after HFNO failure *n* = 233	NIV after HFNO failure (non-intubated patients) *n* = 106	NIV after HFNO failure (intubated patients) *n* = 122	*p*-value
**Exploratory outcomes**				
Mortality at D90, No./total (%)	59/233 (25.3)	2/106 (1.9)	56/122 (45.9)	*p* < 0.001
Length of stay ^a^, median [IQR], d				
In ICU	18.0 [11.0–31.0]	8.0 [5.0-10.2]	21.5 [13.0–32.0]	*p* < 0.001
In Hospital^b^	28.0 [17.0–44.0]	17.0 [11.5–23.0]	34.0 [20.2–54.8]	*p* < 0.001

Abbreviations: HFNO: high flow nasal oxygenation. NIV: noninvasive ventilation. ICU: intensive care unit

^a^ Length of stay were assessed only in patients alive at Day-90 (*n* = 344)

^b^ One missing data (*n* = 343)

Clinical and respiratory parameters of intubated and non-intubated patients in the NIV group were reported in **Additional file 6: Table S4.** We identified the following risk factors to predict endotracheal intubation in the NIV group: age ≥ 57 years old, diabetes mellitus, chronic heart failure, SAPSII score ≥ 26, H2 ROX index under HFNO ≤ 5.7, and PaO2/FiO2 ratio ≤ 110 on the last arterial blood gas under HFNO **(**Table 4**).**

**Table 4:** Exploratory outcomes: unadjusted analysis to predict the risk of endotracheal intubation^a^ in the group NIV after HFNO failure

	No. (%)			
Subgroup	NIV after HFNO failure (non-intubated patients) *n* = 106	NIV after HFNO failure (intubated patients) *n* = 122	OR [IC95%]	p-value
Age group, y				
< 57	39 (62.9)	23 (37.1)	-	-
≥ 57	67 (40.4)	99 (59.6)	2.51 [1.38–4.62]	*p* = 0.003
Diabetes milletus				
No	75 (52.1)	69 (47.9)	-	-
Yes	31 (36.9)	53 (63.1)	1.86 [1.08–3.25]	*p* = 0.027
Chronic heart failure^b^				
No	94 (50.3)	93 (49.7)	-	-
Yes	12 (29.3)	29 (70.7)	2.44 [1.20–5.24]	*p* = 0.017
SAPSII				
< 26	43 (68.3)	20 (31.7)	-	-
≥ 26	63 (38.2)	102 (61.8)	3.48 [1.90–6.55]	*p* < 0.001
H2 ROX index under HFNO				
> 5.7	30 (62.5)	18 (37.5)	-	-
≤ 5.7	62 (40.5)	91 (59.5)	2.45 [1.27–4.84]	*p* = 0.009
PaO2/FiO2 ratio^c^				
> 110	33 (63.5)	19 (36.5)	-	-
≤ 110	59 (41.5)	83 (58.5)	2.44 [1.28–4.77]	*p* = 0.008

Abbreviations: NIV: noninvasive ventilation. HFNO: high flow nasal oxygenation. SAPSII: simplified acute physiology score II. ROX index defined as (SpO2/FiO2)/respiratory rate

^a^ Quantitative variables were then dichotomized using a threshold corresponding to a sensitivity of at least 80% to predict NIV failure in order to better stratify patients at risk of intubation

^b^ Chronic heart failure corresponds to history of coronary artery disease. or documented heart failure in the medical records

^c^ PaO2/FiO2 ratio measured on the last blood arterial gas under HFNO before switch to NIV.

Among subgroups of intubated patients, median time from ICU admission to intubation was 1 day [0–2] for straight intubation group and 3 days [1–6] in NIV group (*p* = 0.036). Duration of IMV was similar in the two groups, 14 days [7–25] vs. 14.5 days [9.5–24] respectively **(Additional file 7: Table S5)**. Among adverse events, the only significant difference was a higher number of VAPs in the straight intubation group, which could be explained by a center effect. **(Additional file 8: Table S6)**.

## Discussion

In this multicenter cohort study conducted in severely hypoxemic COVID-19 patients admitted to ICUs and requiring HFNO, we found a significant reduction of ICU length of stay with no excess 90-day mortality rate between adjusted populations of patients straightly intubated and patients receiving NIV in rescue therapy after HFNO failure. However, there was an increased risk of mortality and length of stay in the subgroup of patients finally intubated after NIV failure.

This study highlights the ethical dilemma between choosing collective benefit over individual risk that clinicians faced during the COVID-19 outbreak [20, 21]. Indeed, 46.5% of patients in the group NIV after HFNO failure were not intubated. This is an interesting result to consider in terms of ICU bed management and burden of care (potential collective benefit). Nevertheless, the subgroup of intubated patients after failure of this strategy paid a heavy price with an increase of mortality and global length of stay (individual risk).

Through the various waves of the epidemic, a better understanding of the pathogenesis of SARS CoV-2 has led to a mortality reduction [22]. In addition, the increasing use of non-invasive strategies (NIV and HFNO) in intermediate care units and ICUs has reduced the overall intubation rate and freed up ICU beds [23, 24]. A recent meta-analysis showed that, noninvasive ventilation with a helmet and HNFO probably reduced mortality, risk of mechanical ventilation, and duration of hospital stay compared to standard oxygen therapy [25]. Noninvasive respiratory supports can be performed outside ICUs and could play a pivotal role to preserve ICU capacity in the global context of future respiratory viral pandemics [26].

Here, we investigated the potential benefit effect of a rescue strategy with additional NIV in case of HFNO failure in severely hypoxemic COVID-19 patients. Urbina et al. reported some advantages of this strategy in a small cohort [27]. This “all noninvasive” strategy is finally the result of the “wait and see” approach advocated by authors such as Papoutsi et al. [15]. However, the question of the ideal timing for intubation remains unresolved [28–30]. To sum up, mortality appears to be higher when either a “very early” or a “very delayed” intubation strategy is used [31].

The selection of patients who could benefit most from noninvasive respiratory strategies remains a major challenge. To help clinicians at the bedside, we identified several risk factors in patients undergoing HFNO among comorbidities, severity score and respiratory parameters to predict failure of endotracheal intubation and NIV strategy. These clinical factors are in line with a previously published nomogram [32] or the HACOR score used to predict failure of non-invasive strategies [33]. Physiological studies have also reported other risk factors such as mechanical power [34], dead space [35], respiratory drive [36] and lung stress [37] that were not available or evaluated in our study. These results showed the need to tailor oxygen support strategies according to patients’ respiratory parameters and comorbidities and to test these risk factors in prospective validation cohorts.

In two large cohorts of COVID-19 patients treated with noninvasive oxygen support, IMV was an independent predictor of lower long-term quality of life and functional outcomes [38, 39]. The authors reported that NIV, when applied using a helmet in strict compliance with the pre-specified criteria for intubation, appeared to be effective and safe. Gonzales et al. showed that a delay superior to 48 h in intubation from the first respiratory support was associated with an increase of in hospital mortality and worse long-term pulmonary sequelae [13]. Here, in the subgroup of patients intubated after failure of HFNO and NIV, median time to intubation was 3 days, which can explain a part of the increased mortality rate (45.9%). These results were also reported in non-covid patients, where a longer duration of NIV before intubation (78 h) was associated with increased mortality [40]. Our safety analysis did not find an increase in ventilation-related complications in intubated patients after NIV failure. Self-inflicted lung injury was another possibility but we did not control esophageal pressure or calculate the lung stress in this study [14]. One possible explanation is that the increased mortality observed in this subgroup is linked to a more severe and longer disease course [41] or a higher severity score and deeper hypoxemic failure, as confirmed by our exploratory results.

Our study has several limitations, mainly due to its retrospective design which limits the strength of the results. First, the decision of straight intubation after HFNO failure or switch to NIV was not standardized. To minimize this bias, a study committee verified from medical records and laboratory tests that this decision was motivated solely by worsening respiratory parameters. The severity of hypoxemia reported in both groups validates the correct selection of patients. In addition, a propensity score was used to balance the two groups on clinical data and respiratory parameters and we chose strict primary and secondary outcomes. Second, some ventilator settings were not available such as tidal volume during NIV sessions, plateau pressure and compliance after intubation to discriminate the natural course of COVID-19 from self-inflicted injuries. There was no protocolized monitoring of the effectiveness of NIV (capnography or pulmonary stress), which may also have contributed to the delay in intubation and explained the increased mortality observed in the subgroup of patients intubated after NIV failure. Third, the type of interface (helmet or facemask) and the duration of NIV session were not reported. Fourthly, there was a wide disparity between centers in the use of intubation, with a majority of VAPs in the NICE center. For this reason, the center effect was included in the statistical model. Fifth, no long-term data have been recorded to determine pulmonary sequelae and the collective benefit of the NIV strategy in terms of quality of life. Finally, due to the lack of data concerning the causes of death, it was not possible to conclude whether the mortality in the NIV group was due to failure of NIV or the natural course of the disease.

Although our study included highly selected patients treated by dexamethasone and HFNO according to the current guidelines, the global mortality rate of the cohort and length of stay were similar to recent publications. The multicenter design, including university and general hospitals is in favor of good generalizability of our results.

## Conclusions

Among severely hypoxemic COVID-19 patients, no significant differences on day-90 mortality were found between patients directly intubated after HFNO failure and patients who received NIV after HFNO failure. A NIV strategy after HFNO failure was associated with a significant reduction in ICU and hospital length of stay, despite an increase in mortality in the subgroup of patients finally intubated. Further studies will need to focus on the role of NIV after HFNO failure, particularly in the event of future pandemics and limited ICU capacity.

### Electronic supplementary material

Below is the link to the electronic supplementary material.

Supplementary Material 1

## Data Availability

No datasets were generated or analysed during the current study.

## Abbreviations

BMI: Body mass index
COVID-19: Coronavirus disease 19
HFNO: High flow oxygen therapy
ICU: Intensive care unit
IMV: Invasive mechanical ventilation
NIV: Noninvasive ventilation
PaO2/FiO2: Pressure of arterial oxygen to fraction of inspired oxygen concentration
ROX index: Respiratory rate oxygenation index
SARS CoV-2: Severe acute respiratory syndrome coronavirus 2
SAPS II: Simplified acuted physiology score II
SOFA score: Sepsis related organ failure assessment score
VAP: Ventilator-associated pneumonia
