# Supplementary material for: Effect of noninvasive ventilation on mortality and clinical outcomes among patients with severe hypoxemic COVID-19 pneumonia after high-flow nasal oxygen failure: a multicenter retrospective French cohort with propensity score analysis
Source: Respir Res. 2024 Jul 15;25:279. doi: 10.1186/s12931-024-02873-4 (PMC11251296; doi:10.1186/s12931-024-02873-4)
Supplement: Supplementary file 1 — Supplementary Material 1 [file 12931_2024_2873_MOESM1_ESM.docx]

**Effect of Noninvasive ventilation on mortality and clinical outcomes among patients with severe hypoxemic COVID-19 pneumonia after high-flow nasal oxygen failure:**

**A multicenter retrospective French cohort with propensity score analysis.**

Antoine GOURY^1^, MD; Zeyneb HOULLA^1^, MD; Mathieu JOZWIAK^2,3^, MD PhD; Tomas URBINA^4^, MD; Matthieu TURPIN^5^, MD; Alexandra LAVALARD^6^, MD; Driss LAGHLAM^7^, MD; Sebastian VOICU^8^, MD, PhD; Jeremy ROSMAN^9^, MD; Claire COUTUREAU^10,11^, MD; Bruno MOURVILLIER^1,12^; MD PhD

^1^ ~~CHU Reims,~~ Unité de Médecine Intensive et Réanimation Polyvalente, CHU Reims, Reims, F-51100 ~~Reims~~, France

^2^ Service de Médecine Intensive Réanimation, Hôpital l’Archet 1, Centre Hospitalier Universitaire de Nice, France

^3^ UR2CA - Unité de Recherche Clinique Côte d'Azur, Université Côte d’Azur, Nice, France

^4^ Service de Médecine Intensive Réanimation, Hôpital Saint-Antoine, Assistance Publique Hôpitaux de Paris, Paris, France

^5^ Service de Médecine Intensive Réanimation, Hôpital Tenon, Assistance Publique Hôpitaux de Paris, Paris, France

^6^ Unité de Réanimation Polyvalente, Centre Hospitalier de Troyes, Troyes, France

^7^ Service de Médecine Intensive et Réanimation, Hôpital Cochin, Assistance Publique Hôpitaux de Paris, Paris, France

^8^ Réanimation Médicale et Toxicologique, Hôpital Lariboisière, Assistance Publique Hôpitaux de Paris, Paris, France

^9^ Unité de Soins Intensifs et Réanimation ~~Service de Médecine Intensive Réanimation~~, Centre Intercommunale Nord-Ardennes, Charleville-Mézières, France

^10^ Université de Reims Champagne-Ardenne, VieFra, F-51100 Reims, France

^11^ ~~CHU Reims,~~ Unité d’Aide Méthodologique, CHU Reims, Reims, F-51100 ~~Reims~~, France

^12^ Université de Reims Champagne-Ardenne, EA-4684 CardioVir, Reims, F-51100 ~~Reims~~, France

***Corresponding author:***

Antoine GOURY, M.D.

Intensive care Department, Reims University Hospitals, Reims, France

**5 Rue du general Koenig, 51100 Reims, France**

**Tel : (+33) 6 80 63 36** [21](C:\\Users\\FiercePC\\Downloads\\21  Orcid: https:\\orcid.org\\0000-00)

[Orcid: https://orcid.org/0000-00](C:\\Users\\FiercePC\\Downloads\\21  Orcid: https:\\orcid.org\\0000-00)01-7888 8570

**e-mail :** [agoury@chu-reims.fr](mailto:bschwartz@chu-reims.fr)

**Additional figures legends**

**Additional file 3: Fig S1:** Covariate balance measured by standardized mean difference

1. Before and after weighting
2. Before and after matching

The effect of NIV on 90 days mortality and the ICU and hospital length of stay were assessed using a propensity score analysis to control confounding factors between groups. The probability of receiving NIV was calculated using logistic regression. Covariates included in the propensity score model were confounders and variables related to the outcome (i.e. 90 days mortality) (18). Thus, the propensity score model including the following comorbidities; sex, age, body mass index (BMI), hypertension, diabetes, chronic heart failure, chronic respiratory diseases, chronic renal failure, immunocompromised state and solid cancers. Moreover, severity markers such as the SAPS II score at admission, the percentage of pulmonary infiltrate on the first chest CT-scan, the ROX index at H2 with HFNO and the last PaO2/FiO2 ratio before intubation or switch to NIV were also included. Then, a weighted logistic regression with stabilized inverse probability of treatment weighting (IPTW) was performed, adjusted for center. Weights were truncated at the 1^st^ and 99^th^ percentiles to control extreme weights. A sensitivity analysis was performed with propensity score matching on a 1:1 ratio using a caliper of 0.1. To deal with missing data, multiple imputations by chained equations were performed. Twenty imputed data sets were generated after 25 iterations. Propensity score analyses were performed on these 20 complete data sets using a within approach. We assessed balance between groups on the chosen variables before and after propensity score matching or weighting using standardized mean differences from complete imputed data sets.

**Additional file 5: Fig S2:** Comparison of Kaplan-Meier survival curves between straight intubation group, NIV after HFNO failure (intubated patients) group, and NIV after HFNO failure (non-intubated patients) groups.

Abbreviations: HFNO: high flow nasal oxygenation. NIV: noninvasive ventilation.

**Additional file 1: Table S1:** List of participating centers with proportion of patients included in the final analysis.

|  | **Straight intubation after HFNO failure**  **(n=233)** | **NIV after HFNO failure**  **(n=228)** |
| --- | --- | --- |
| **Centers** | **n (%)** | **n (%)** |
| Charleville-Mézières | 4 ( 1.7) | 4 ( 1.8) |
| Cochin | 35 (15.0) | 13 ( 5.7) |
| Lariboisière | 2 ( 0.9) | 27 (11.8) |
| Nice | 109 (46.8) | 18 ( 7.9) |
| Reims | 26 (11.2) | 55 (24.1) |
| St Antoine-Tenon | 52 (22.3) | 41 (18.0) |
| Troyes | 5 ( 2.1) | 70 (30.7) |

Abbreviations: HFNO: high flow nasal oxygen. NIV: noninvasive ventilation.

**Additional file 2: Table S2:** STROBE checklist.

|  | Item No. | Recommendation | Page  No. |
| --- | --- | --- | --- |
| **Title and abstract** | 1 | (*a*) Indicate the study’s design with a commonly used term in the title or the abstract | 1 |
|  |  | (*b*) Provide in the abstract an informative and balanced summary of what was done and what was found | 2 |
| Introduction | | | |
| Background/rationale | 2 | Explain the scientific background and rationale for the investigation being reported | 4 |
| Objectives | 3 | State specific objectives, including any prespecified hypotheses | 4 |
| Methods | | | |
| Study design | 4 | Present key elements of study design early in the paper | 5 |
| Setting | 5 | Describe the setting, locations, and relevant dates, including periods of recruitment, exposure, follow-up, and data collection | 5 |
| Participants | 6 | Give the eligibility criteria, and the sources and methods of selection of participants. Describe methods of follow-up | 5 |
| Variables | 7 | Clearly define all outcomes, exposures, predictors, potential confounders, and effect modifiers. Give diagnostic criteria, if applicable | 6 |
| Data sources/ measurement | 8* | For each variable of interest, give sources of data and details of methods of assessment (measurement). Describe comparability of assessment methods if there is more than one group | 6 |
| Bias | 9 | Describe any efforts to address potential sources of bias | 7 |
| Study size | 10 | Explain how the study size was arrived at | Not applicable |

| Quantitative variables | 11 | Explain how quantitative variables were handled in the analyses. If applicable, describe which groupings were chosen and why |  |
| --- | --- | --- | --- |
| Statistical methods | 12 | (*a*) Describe all statistical methods, including those used to control for confounding | 6,7 Additional file 3: Figure S1. |
|  |  | (*b*) Describe any methods used to examine subgroups and interactions | 6,7 Additional file 3, Figure S1 |
|  |  | © Explain how missing data were addressed | 7 |
|  |  | (*d*) *Cohort study*—If applicable, explain how loss to follow-up was addressed | - |
|  |  | (*e*) Describe any sensitivity analyses | 7 |
| Participants | 13* | (a) Report numbers of individuals at each stage of study—eg numbers potentially eligible, examined for eligibility, confirmed eligible, included in the study, completing follow-up, and analysed | Figure 1 : Flowchart |
|  |  | (b) Give reasons for non-participation at each stage | Figure 1 : Flowchart |
|  |  | (c) Consider use of a flow diagram | Figure 1 : Flowchart |
| Descriptive data | 14* | (a) Give characteristics of study participants (eg demographic, clinical, social) and information on exposures and potential confounders | Table 1 |
|  |  | (b) Indicate number of participants with missing data for each variable of interest | Table 1 |
|  |  | (c) *Cohort study*—Summarise follow-up time (eg, average and total amount) | - |
| Outcome data | 15* | *Cohort study*—Report numbers of outcome events or summary measures over time | Table 2, Figure 2 |

**Results**

| Main results |  | (*a*) Give unadjusted estimates and, if applicable, confounder-adjusted estimates and their precision (eg, 95% confidence interval). Make clear which confounders were adjusted for and why they were included | 7,8 |
| --- | --- | --- | --- |
| Other analyses | 17 | (*b*) Report category boundaries when continuous variables were categorized | 9 |
| **Discussion** |  |  |  |
| Key results | 18 | (*c*) If relevant, consider translating estimates of relative risk into absolute risk for a meaningful time period | 9 |
| Limitations | 19 | Discuss limitations of the study, taking into account sources of potential bias or imprecision. Discuss both direction and magnitude of any potential bias | 9,10,11 |
| Interpretation | 20 | Give a cautious overall interpretation of results considering objectives, limitations, multiplicity of analyses, results from similar studies, and other relevant evidence | 9, 10, 11 |
| Generalisability | 21 | Discuss generalizability (external validity) of the study results | 11 |
| Other information | |  |  |
| Funding | 22 | Give the source of funding and the role of the funders for the present study and, if applicable, for the original study on which the present article is based | 19 |

|  | **All patients** | **Straight intubation after HFNO failure**  **(n=233)** | **NIV after HFNO failure (intubated patients)**  **(n= 122)** | **NIV after HFNO failure (non-intubated patients)**  **(n= 106)** |
| --- | --- | --- | --- | --- |
| **Reasons to switch to NIV** |  |  |  |  |
| Poor oxygenation/desaturation | 205/228 (90) | - | 117 (96) | 88 (83) |
| Intolerance/agitation | 23/228 (10) | - | 5 (4) | 18 (17) |
| **Reasons to intubation** |  |  |  | - |
| Poor oxygenation/desaturation | 301/355 (85) | 192 (82.5) | 109 (89) | - |
| Intolerance/agitation | 40/355 (11) | 33 (14) | 7 (6) | - |
| Other^a^ | 14/355 (4) | 8 (3.5) | 6 (5) | - |

**Additional file 4. Table S3:** Reasons to switch HFNO to NIV or intubation according to the groups.

Abbreviations: NIV: noninvasive ventilation; HFNO: High flow oxygen therapy

^a^Other: cardiac arrest n=6 in the group NIV after HFNO failure (intubated patients) and n=5 in the group Straight intubation after HFNO failure. Hemodynamic instability n=3 in the group Straight intubation after HFNO failure.

**Additional file 6: Table S4:** Exploratory endpoints: Unadjusted subgroup analyses

|  | NIV after HFNO failure (non-intubated patients)  (n= 106) | NIV after HFNO failure (intubated patients)  (n= 122) | p-value | Missing data (n) |
| --- | --- | --- | --- | --- |
| Age. median [IQR], y | 61 [51-72] | 64 [58-71] | 0.092 | - |
| Males, n (%) | 69 (65) | 87 (71) | 0.314 | - |
| BMI. median [IQR], kg.m² | 31 [26-35] | 31 [27-35] | 0.458 | 3 |
| SOFA score at admission, median [IQR] | 3 [2-4] | 4 [4-5] | <0.001 | - |
| SAPSII, median [IQR] | 28 [22-33] | 33 [27-42] | <0.001 | - |
| Time from, median [IQR], d |  |  |  |  |
| Symptoms onset to hospital admission | 7 [5-8] | 7 [4-9] | 0.500 | - |
| HFNO duration^a^, median [IQR], d | 6 [4-9] | 2 [1-6] | <0.001 | 2 |
| NIV duration, median [IQR], d | 3 [1-6] | 2 [1-4] | <0.001 | 2 |
| Comorbidities, n (%) |  |  |  |  |
| Hypertension | 53 (50) | 75 (62) | 0.082 | - |
| Diabetes mellitus | 31 (29) | 53 (43) | 0.027 | - |
| Chronic heart failure^b^ | 12 (11) | 29 (24) | 0.015 | - |
| Chronic lung disease^c^ | 16 (15) | 12 (10) | 0.228 | - |
| Chronic kidney disease^d^ | 5 (5) | 8 (7) | 0.550 |  |
| Immunocompromised state^e^ | 9 (9) | 13 (11) | 0.581 | - |
| Solid cancer | 6 (6) | 8 (7) | 0.778 | - |
| First chest CT scan findings |  |  |  |  |
| Percentage of pulmonary infiltrates, median [IQR] | 50 [37-62] | 62 [37-71] | 0.212 | 13 |
| Respiratory findings at H2 of HFNO, median [IQR] |  |  |  |  |
| Oxygen saturation, % | 95.0 [93.0-97.0] | 93.3 [91.0-96.0] | 0.002 | 12 |
| FiO2, % | 80.0 [60.0-100.0] | 90.0 [70.0-100.0] | 0.003 | 13 |
| Respiratory rate, breaths/min | 25.0 [21.0-33.0] | 27.0 [22.0-31.0] | 0.247 | 24 |
| ROX index | 4.6 [3.8-6.3] | 4.1 [3.4-5.2] | 0.004 | 27 |
| First arterial blood gas with HFNO^f^, median [IQR] |  |  |  |  |
| FiO2, % | 80 [62-100] | 90 [70-100] | 0.006 | 14 |
| PaO2, mmHg | 73.3 [62.2-87.2] | 70.0 [60.9-89.5] | 0.848 | 15 |
| PaCO2, mmHg | 35.0 [32.0-38.3] | 35.0 [31.9-38.5] | 0.860 | 15 |
| PaO2/FiO2 ratio | 92.8 [73.0-123.1] | 83.9 [69.0-110.5] | 0.096 | 15 |
| Last arterial blood gas with HFNO^g^, median [IQR] |  |  |  |  |
| FiO2, % | 80 [70-100] | 92 [80-100] | 0.003 | 33 |
| PaO2, mmHg | 73.0 [64.0-93.0] | 66.0 [57.0-82.5] | 0.009 | 32 |
| PaCO2, mmHg | 34.0 [31.0-37.4] | 35.4 [32.0-39.0] | 0.282 | 32 |
| PaO2/FiO2 ratio | 96.2 [78.7-127.9] | 80.6 [61.8-101.7] | <0.001 | 32 |

Abbreviations: HFNO: high flow nasal oxygen. NIV: noninvasive ventilation.

^a^ In the NIV group, HFNO was administrated between NIV sessions for patients receiving intermittent NIV.

^b^ Chronic heart failure corresponds to history of coronary artery disease. or documented heart failure in the medical records.

^c^ Chronic obstructive pulmonary disease or obstructive sleep apnea.

^d^ Chronic kidney disease corresponds to a KDIGO stage 3 and more.

^e^ Immunocompromised state corresponds to patients with hematologic malignancies. treatment based on corticosteroids or immunosuppressive therapies.

^f^ the closest to the introduction of the HFNO and within 12 hours maximum.

^g^ the closest to switch to NIV and within the 12 last hours with HFNO.

**Additional file 7: Table S5:** Exploratory endpoints: clinical outcomes for patients who required endotracheal intubation.

|  | **Straight intubation after HFNO failure (n=233)** | **NIV after HFNO failure (intubated patients)**  **(n=122)** | **p-value** | **Missing data**  **(n)** |
| --- | --- | --- | --- | --- |
| Time from ICU admission to intubation, median [IQR], d | 1 [0-2] | 3 [1-6] | 0.036 | - |
| Last arterial blood gas before intubation, median [IQR] |  |  |  |  |
| FiO2, % | 100 [80-100] | 100 [90-100] | 0.090 | 20 |
| PaO2, mmHg | 65 [55-76] | 67 [59-75] | 0.450 | 14 |
| PaCO2, mmHg | 33 [29-37] | 37 [34-40] | <0.001 | 14 |
| PaO2/FiO2 ratio | 71 [60-87] | 71 [60-84] | 0.918 | 20 |
| Duration of IMV, median [IQR], d | 14.0 [7.0-25.0] | 14.5 [9.5-24] | 0.352 | - |
| Ventilator-associated pneumonia^a^ | 159 (68.2) | 58 (47.5) | <0.001 | - |
| Barotrauma^b^ | 8 (5.6) | 7 (7.3) | 0.586 | 115 |
| Cardiac arrest | 5 (3.4) | 6 (6.2) | 0.352 | 113 |
| Renal replacement therapy | 38 (16.3) | 15 (12.3) | 0.314 | - |
| Pulmonary embolism | 3 (1.7) | 5 (4.7) | 0.150 | 68 |
| Prone positioning | 59 (25.3) | 29 (23.6) | 0.748 | - |
| Nitric oxide | 34 (18.8) | 30 (28.6) | 0.056 | 69 |
| Neuromuscular blockade | 214 (92.2) | 111 (92.5) | 0.931 | 3 |
| Extracorporeal membrane oxygenation | 7 (3.0) | 4 (3.3) | 1.000 | - |

Abbreviations: NIV: noninvasive ventilation; HFNO: High flow oxygen therapy; IMV: invasive mechanical ventilation.

^a^ Ventilator-associated pneumonia were reported by clinician in each center. Diagnosis of ventilator-associated pneumonia was not standardized.

^b^ Barotrauma included pneumothorax and pneumomediastinum.

**Additional file 8: Table S6:** Number of ventilator-associated pneumonia (VAP) reported per center among intubated patients.

| **Centers** | **No VAP**  **n=138** | **VAP**  **n=217** |
| --- | --- | --- |
| Charleville-Mézières | 1 (0.7) | 3 (1.4) |
| Cochin | 11 (8.0) | 34 (15.7) |
| Lariboisière | 1 (0.7) | 12 (5.5) |
| Nice | 13 (9.4) | 107 (49.3) |
| Reims | 27 (19.6) | 26 (12.0) |
| St Antoine-Tenon | 46 (33.3) | 22 (10.1) |
| Troyes | 39 (28.3) | 13 (6.0) |

The Nice-based center reported a higher rate of VAP than the other centers.

**
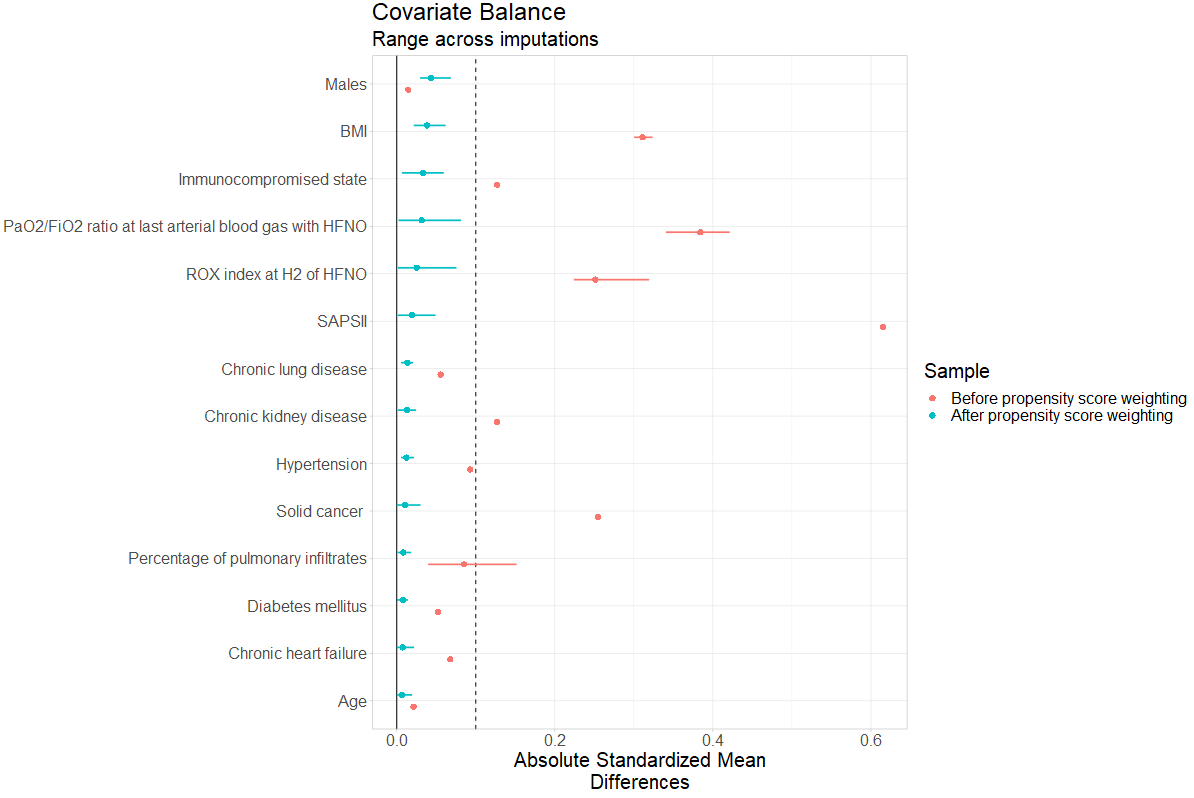
**

**B)**

**
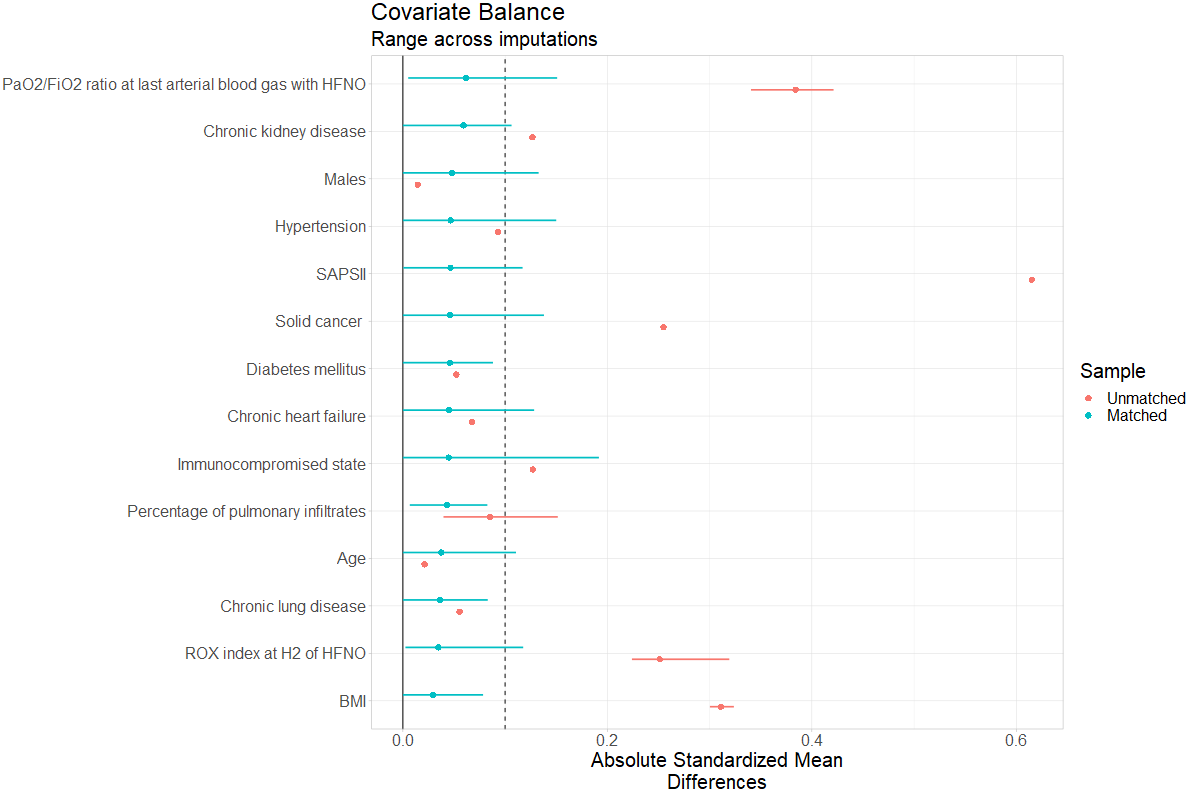
**

**Additional file 3: Fig S1:**


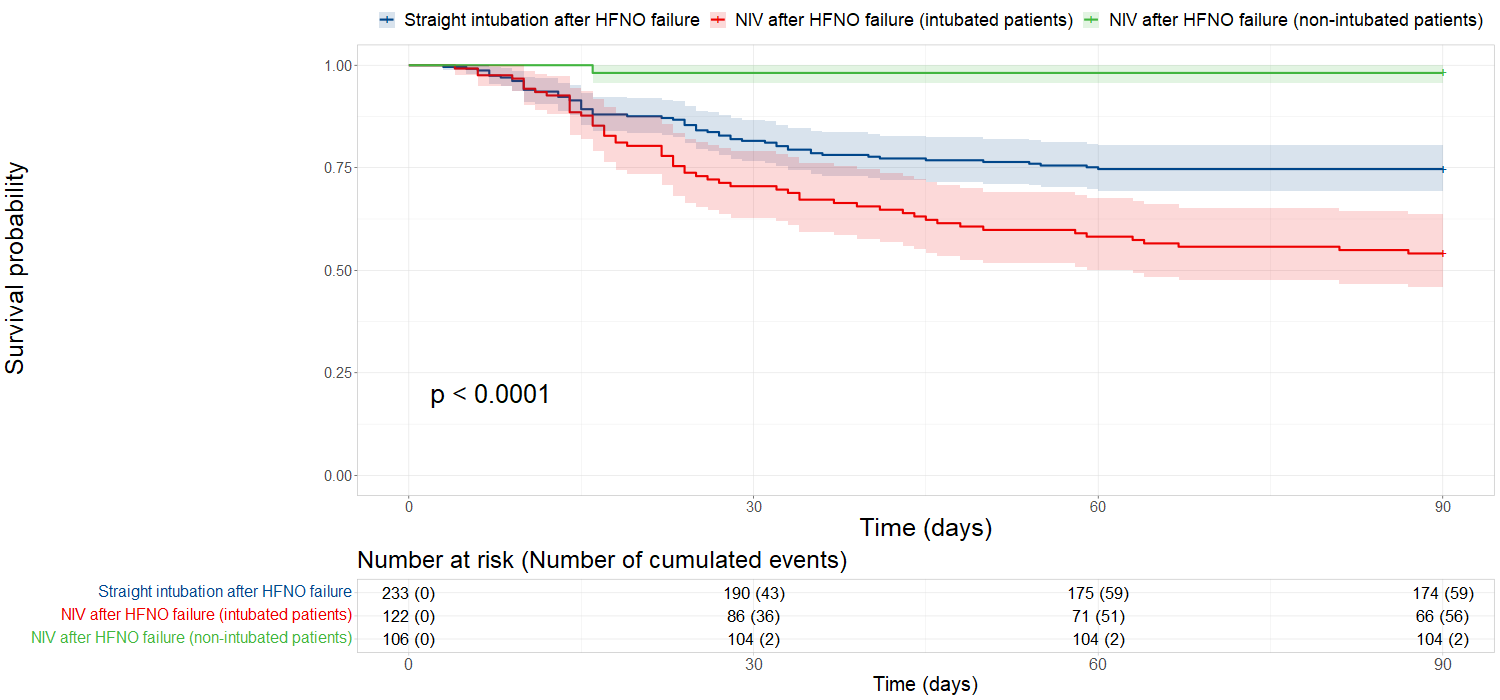


**Additional file 5: Fig S2.**
